# Epidemiological Survey of Dyslipidemia in Civil Aviators in China from 2006 to 2011

**DOI:** 10.1155/2014/215076

**Published:** 2014-02-17

**Authors:** Rongfu Zhao, Dan Xiao, Xiaoying Fan, Zesong Ge, Linsheng Wang, Tiecheng Yan, Jianzhi Wang, Qixin Wei, Yan Zhao

**Affiliations:** ^1^Xi'an Civil Aviation Hospital, Xi'an 710082, China; ^2^Department of Epidemiology and Ministry of Education Key Lab of Hazard Assessment and Control in Special Operational Environment, School of Public Health, Fourth Military Medical University, No. 169, Changle West Road, Xi'an 710032, China; ^3^Xijing Hospital, Fourth Military Medical University, Xi'an 710032, China; ^4^China Eastern Airlines, Xi'an 710082, China; ^5^Hainan Airlines, Xi'an 710082, China

## Abstract

*Aim*. This study aimed to analyze blood lipid levels, temporal trend, and age distribution of dyslipidemia in civil aviators in China. *Methods*. The 305 Chinese aviators were selected randomly and followed up from 2006 to 2011. Their total cholesterol (TC), triglyceride (TG), high-density lipoprotein cholesterol (HDL-C), and low-density lipoprotein cholesterol (LDL-C) levels were evaluated annually. Mean values for each parameter by year were compared using a linear mixed-effects model. The temporal trend of borderline high, high, and low status for each index and of overall borderline high, hyperlipidemia, and dyslipidemia by year was tested using a generalized linear mixed model. *Results*. The aviators' TC (*F* = 4.33, *P* < 0.01), HDL-C (*F* = 23.25, *P* < 0.01), and LDL-C (*F* = 6.13, *P* < 0.01) values differed across years. The prevalence of dyslipidemia (*F* = 5.53, *P* < 0.01), borderline high (*F* = 6.52, *P* < 0.01), and hyperlipidemia (*F* = 3.90, *P* < 0.01) also differed across years. The prevalence rates for hyperlipidemia and dyslipidemia were the highest in the 41–50-year-old and 31–40-year-old groups. *Conclusions*. Civil aviators in China were in high dyslipidemia and borderline high level and presented with dyslipidemia younger than other Chinese populations.

## 1. Introduction

Dyslipidemia is an important risk factor for cardiovascular disease, which is the leading cause of morbidity and mortality worldwide and a major contributor to the global burden of disease [1–3]. With the development of the economy and increase in average living standards in China, there has been a trend of increasing prevalence of dyslipidemia, which has, as a result, become a serious health threat in China [4, 5]. As of 2005, the prevalence of dyslipidemia in Chinese adults had reached 18.6%, which equates to approximately 16 billion people [5].

Because of their special working environment, dyslipidemia may be more harmful to aviators than to people in the general populations. Dyslipidemia may lead to aerorelated diseases, such as vertigo, syncope, and flight illusion, which could be dangerous during flight. Indeed, nearly half of grounded aviators are grounded due to cardiovascular disease, a condition which correlates with dyslipidemia [6]. The sudden onset of cardiovascular symptoms during flight may have serious, potentially life-threatening consequences on passengers and crew. However, because there are not obvious early signs of dyslipidemia, people tend to overlook dyslipidemia relative to other conditions with more discernible clinical signs and symptoms. Consequently, prevention and treatment of dyslipidemia tend to be overlooked.

Blood lipid profiles differ across populations, regions, and time periods due, at least in part, to ethnicity factors, working environment, geography, and lifestyle [7–9]. Indeed, the characteristics of dyslipidemia in Chinese population as high TG and low HDL-C are different from that of people in Western countries as high TC [5]. And the few studies in the literature addressing dyslipidemia of civil aviators in China have reported prevalence rates ranging from 51.4% to 68.7% [10, 11], which were higher than those in the general Chinese population [12–15]. Compared with the general Chinese population, civil aviators in China consume a diet that is higher in fat and do less physical exercises, which may increase risk of dyslipidemia. However, studies on dyslipidemia of civil aviators in China were all cross-sectional surveys which cannot detect the temporal trend of dyslipidemia in aviators. Therefore, the temporal trends and distribution among civil aviators in China remain unclear factors which hinder the implementation of an effective control and prevention strategies for this special population. There is a need to clarify the current situation of dyslipidemia among civil aviators in China.

The principal aim of this study was to explore blood lipid levels, prevalence, temporal trends, and age distribution of dyslipidemia among civil aviators in China. The resultant data can provide a scientific basis for understanding the current situation of dyslipidemia and for the formulation and implementation of targeted control and prevention measures for civil aviators residing in China.

## 2. Materials and Methods

### 2.1. Ethics Statement

This study was approved by the Ethics Committee of Xi'an Civil Aviation Hospital (Grant no. 200506). All participants provided their written consent to participate in this study.

### 2.2. Study Population

All civil aviators based in China are obligated to receive an annual physical examination between August and October to obtain an aviator-requisite health certificate. A group of 305 were chosen randomly by random sampling from all aviators who received a physical examination in 2006 in an aviation medicine examination center in Xi'an Civil Aviation Hospital. All were male civil aviators, 20–55 years old, and with a cumulative flight time greater than 200 hours. All 305 participants were followed up from 2006 to 2011.

### 2.3. Data Collection and Measurements

The aviators' annual physical examination results were collected by several aviation medicine examination centers and recorded in China's Civil Aviation Medical Certificate Management System that was hosted in Civil Aviation Administration of China. Each aviation medicine examination center can view and analyze the data of aviators who received physical examination in their hospital. All data used in this study were collected from this system by the aviation medicine examination center in Xi'an Civil Aviation Hospital and represented results collected from 2006 to 2011, including subject height, weight, systolic blood pressure (BP), diastolic BP, pulse, total cholesterol (TC), triglyceride (TG), high-density lipoprotein cholesterol (HDL-C), and low-density lipoprotein cholesterol (LDL-C) levels. Body mass index (BMI) was calculated for each subject according to the standard formula: BMI = weight (kg)/[height (m)]^2^. All data was anonymized before it was provided to the study group.

Greasy food (i.e., high-fat and fried foods) was banned for the 3 days before each physical examination. Blood samples were obtained from forearm venipuncture when the civil aviators had an empty stomach in the morning (8:00–10:00 am). The blood samples were centrifuged at 4°C and 3,000 rpm for 10 minutes and the serum was transferred and stored at −20°C for biochemical analyses. TC, TG, HDL-C, and LDL-C were measured enzymatically using the COBAS INTEGRA 400 Plus system and its affiliated reagents from Roche Company (USA). All tests were regular examination items in the civil aviator physical examination.

### 2.4. Cut-Off Points for Blood Lipid Indices

The cut-off points for blood lipid indices were defined according to the criteria set forth in the guidelines for dyslipidemia control in Chinese adults (2007 edition) [16]. TC was defined as normal, borderline high (+), or high (++) if it was in the range of <5.18 mmol/L, 5.18–6.19 mmol/L, and ≥6.22 mmol/L, respectively. TG was defined as normal, borderline high, or high if it was in the range of <1.70 mmol/L, 1.70–2.25 mmol/L, and ≥2.26 mmol/L, respectively. LDL-C was defined as normal, borderline high, or high if it was in the range of <3.37 mmol/L, 3.37–4.12 mmol/L, and ≥4.14 mmol/L, respectively. HDL-C was defined as low if it was ≤1.04 mmol/L. Total blood lipid level was defined as borderline high if TC, TG, or LDL-C were borderline high. Total blood lipid level was defined as hyperlipidemia if TC, TG, or LDL-C were high. If a civil aviator presented a high grade for any index, he was categorized as having hyperlipidemia, regardless of the other blood lipid index values. If a civil aviator presented borderline high or hyperlipidemia in terms of total blood lipid level, or had a low HDL-C level, he was also categorized as having dyslipidemia.

### 2.5. Statistical Analysis

The study populations were categorized into the following four age bands (in years): 21–30, 31–40, 41–50, and 50–60. Subjects were not locked in their original age bands but rather were moved from younger to older age band categories as appropriate with aging over time. The mean and standard deviation (SD) of each blood lipid index were calculated for each year, and then the values were compared based on a linear mixed-effects model. The prevalence of borderline high, high, and low classification for each blood lipid index and of overall borderline high, hyperlipidemia, and dyslipidemia was calculated for age band and year and then analyzed with a generalized linear mixed model. The linear mixed-effects model and generalized linear mixed model were constructed with age as a covariate. Differences and trends were considered significant at *P* < 0.05. These analyses were performed in SAS 9.2 software.

## 3. Results

### 3.1. Demographic Characteristics of Civil Aviators

The mean age of the 305 subjects studied was 35.52 ± 7.87 in 2006 (minimum 20; maximum 50). Their cumulative flight time at that time ranged from 200 to 25,800 hours, with the mean flight time in 2006 being 10,011.4 hours. The subjects' anthropomorphic, BP, and pulse data are reported in Table 1.

### 3.2. Blood Lipid Levels of Civil Aviators by Year

As reported in detail in Table 2, we observed a significant effect of the passage of time (examination year) on TC, HDL-C, and LDL-C (all *P*s < 0.01) but not on TG. The lowest TC, HDL-C, and LDL-C values were observed in 2007, 2011, and 2007, respectively, and the highest values of these three indices occurred in 2009, 2006, and 2010, respectively (Table 2). As shown in Table 3, from 2006 to 2011, there was an increasing trend for TC values (fixed coefficients of linear mixed-effects model during 2006–2010 versus 2011 were −0.03, −0.29, −0.01, 0.10, and 0.02) and a decreasing trend for HDL-C values (fixed coefficients of linear mixed-effects model during 2006–2010 versus 2011 were 0.33, 0.14, 0.10, 0.05, and 0.10).

### 3.3. Dyslipidemia and Hyperlipidemia Rate of Civil Aviators by Year

The prevalence rates of overall borderline high, hyperlipidemia, and dyslipidemia differed across years (all *P*s < 0.01). The lowest prevalence rates of overall borderline high, hyperlipidemia, and dyslipidemia were observed in 2011, 2006, and 2006, respectively and the highest values for these three indices occurred in 2006, 2009, and 2009, respectively (Table 4). From 2006 to 2011, increasing trends were observed for prevalence of hyperlipidemia (fixed coefficients of generalized linear mixed model from 2006 to 2010 versus that in 2011 were 0.15, 0.08, 0.50, 0.94, and 0.82) and dyslipidemia (fixed coefficients of generalized linear mixed model from 2006 to 2010 versus that in 2011 were −0.33, −0.28, −0.19, 0.66, and 0.61) (Table 5). The yearly values are reported in Table 4.

### 3.4. Dyslipidemia and Hyperlipidemia Rate of Civil Aviators by Age Group

The prevalence rates for high TC, hyperlipidemia, and borderline high TC, TG, and LDL-C were the highest in the 41–50-year-old group, and the prevalence rates for high TG, low HDL-C, and dyslipidemia were the highest in the 31–40-year-old group, whereas those for high LDL-C and overall borderline high were the highest in the 51–60-year-old group. The prevalence rates of each index in each age group are reported in Table 6.

## 4. Discussion

The present data indicate that, from 2006 to 2011, the prevalence of high TG and of high LDL-C among civil aviators in China was similar to that in the general population in China as a whole [17]. However, the overall blood lipid levels and dyslipidemia prevalence rates in this cohort were higher than those in the general male population in China [4, 7]. These results indicate that dyslipidemia among civil aviators in China is a serious issue and that, consequently, more attention should be paid to the control and prevention of dyslipidemia in this population.

The prevalence of borderline high classifications for the blood lipid indices of civil aviators in China was higher than that of military aviators, whereas the prevalence of high classification for the indices in our cohort was lower than that of military aviators [10]. These differences may be due, at least in part, to the greater flight intensity of military aviators compared with civil aviators, which may elevate the risk of hyperlipidemia for military aviators. Conversely, higher blood lipids among civil aviators could be due to the fact that because they do not live together in groups like military aviators do; their diet and physical exercise are more difficult to regulate. The relatively high prevalence of borderline high indices in our cohort implies that many civil aviators in China are at risk of hyperlipidemia.

High TC and high TG are most prevalent among 45–59-year-old men in China [4] and similarly are most prevalent among 41–45- and 51–55-year-old male military aviators [13]. In this study, we found that high TC and hyperlipidemia were most prevalent in the 41–50-year-old age group, and high TG and dyslipidemia were most prevalent in the 31–40-year-old age group, indicating that civil aviators in China may tend to develop dyslipidemia at younger ages than the general population or military aviators in China. This difference is probably a consequence of their high fat and protein intake compared with the general population and their lesser physical exercise compared with military aviators.

It should be noted that the number of civil aviators recruited in this study is less than that of some large-scale surveys on blood lipid levels because our target population is quite small relative to the general population and we focused on a relatively short study time period. And there may be some other causes of dyslipidemia in aviators such as hypothyroidism, renal disease, hepatic disease, and diabetes. Nevertheless, there was sufficient power in this study to demonstrate significant trends in blood lipid levels, prevalence rates, temporal trends, and age of dyslipidemia onset among civil aviators in China. Further study with more civil aviators and a longer follow-up period will provide more insight into the epidemiology of dyslipidemia among civil aviators in China.

In conclusion, the prevalence of dyslipidemia, especially the prevalence of the borderline high status of each blood lipid index, was high in this population. Civil aviators in China have a propensity to develop dyslipidemia at a younger age than the general population in China and military aviators. There is a need to step up dyslipidemia control and prevention efforts and to adjust public health resource allocation more effectively for the benefit of civil aviators in China.

## Figures and Tables

**Table 1:** Anthropomorphic and cardiovascular characteristics of civil aviators in China in 2006 (*N* = 305).

Parameter	Mean ± SD
Height (cm)	174.73 ± 4.79
Weight (kg)	74.50 ± 7.81
BMI (kg/m^2^)	24.41 ± 2.44
SBP (mmHg)	120.86 ± 15.21
DBP (mmHg)	80.66 ± 6.12
Sphygmus (beats/min)	76.36 ± 7.88

**Table 2:** Blood lipid data for civil aviators in China by year 2006–2011 (*N* = 305).

Year	TC, mmol/L			TG, mmol/L			HDL-C, mmol/L			LDL-C, mmol/L		
	$\overset{-}{x}$	SD	*F*	$\overset{-}{x}$	SD	*F*	$\overset{-}{x}$	SD	*F*	$\overset{-}{x}$	SD	*F*
2006	4.57	0.80	4.33**	1.49	0.73	0.80	1.42	0.30	23.25**	3.29	0.73	6.13**
2007	4.31	0.72		1.48	0.71		1.22	0.26		2.95	0.67	
2008	4.61	0.81		1.60	0.74		1.19	0.24		3.03	0.74	
2009	4.70	0.85		1.54	0.67		1.13	0.25		3.00	0.90	
2010	4.62	0.82		1.57	0.77		1.19	0.26		3.31	0.84	
2011	4.60	0.78		1.47	0.66		1.09	0.25		2.99	0.66	

**P < 0.01.

**Table 3:** Fixed coefficients of each blood lipid index of Chinese civil aviators in linear mixed-effect model by year 2006–2011 (*N* = 305).

Year	TC	TG	HDL-C	LDL-C
Intercept	4.60**	1.47**	1.09**	2.99**
2006	−0.03	0.01	0.33**	0.29**
2007	−0.29**	0.01	0.14**	−0.04
2008	−0.01	0.13	0.10**	0.04
2009	0.10	0.06	0.05	0.01
2010	0.02	0.10	0.10**	0.32**
2011	0	0	0	0

**P < 0.01.

**Table 4:** Lipid index classifications and prevalence of lipid conditions of civil aviators in China by year 2006–2011 (*N* = 305).

Year	TC, *N* (%)		TG, *N* (%)		HDL-C, *N* (%)	LDL-C, *N* (%)		Borderline high, *N* (%)	Hyperlipidemia, *N* (%)	Dyslipidemia, *N* (%)
	+	++	+	++	−	+	++			
2006	55 (18.03)	7 (2.30)	61 (20.00)	42 (13.77)	22 (7.21)	107 (35.08)	26 (8.52)	107 (35.08)	72 (23.61)	180 (59.02)
2007	31 (10.16)	2 (0.66)	50 (16.39)	44 (14.43)	83 (27.21)	68 (22.30)	15 (4.92)	82 (26.89)	123 (40.33)	182 (59.67)
2008	59 (19.34)	11 (3.61)	59 (19.34)	59 (19.34)	81 (26.56)	72 (23.61)	20 (6.56)	75 (24.59)	127 (41.64)	186 (60.98)
2009	68 (22.30)	13 (4.26)	53 (17.38)	48 (15.74)	116 (38.03)	52 (17.05)	39 (12.79)	69 (22.62)	178 (58.36)	224 (73.44)
2010	59 (19.34)	7 (2.30)	61 (20.00)	39 (12.79)	96 (31.48)	85 (27.87)	55 (18.03)	85 (27.87)	149 (48.85)	222 (72.79)
2011	37 (12.13)	13 (4.26)	37 (12.13)	44 (14.43)	140 (45.90)	61 (20.00)	15 (4.92)	61 (20.00)	164 (53.77)	195 (63.93)

*F*	6.95**	1.76	1.47	2.30*	5.22**	4.23**	8.37**	6.52**	3.90**	5.53**

Ranges for all classifications are defined in Methods.

*P < 0.05, **P < 0.01.

**Table 5:** Fixed coefficients of each lipid parameter in Chinese civil aviators in generalized linear mixed model 2006–2011 (*N* = 305).

Year	TC		TG		HDL-C	LDL-C		Borderline high	Hyperlipidemia	Dyslipidemia
	+	++	+	++	−	+	++			
Intercept	−2.94**	−3.60**	−2.39**	−2.81**	−1.92	−1.59**	−3.26**	−1.06**	−2.26**	0.81**
2006	0.68	−0.82	0.71*	−0.10	−3.52**	0.89**	0.66	1.05**	0.15	−0.33
2007	−0.30	−1.95	0.42	−0.00	−1.14**	0.15	0.00	0.45	0.08	−0.28
2008	0.83**	−0.35	0.65*	0.61*	−1.21**	0.20	0.58	0.56*	0.50	−0.19
2009	1.11**	0.00	0.48	0.19	−0.43	−0.22	1.07*	0.86**	0.94**	0.66*
2010	0.83**	−0.93	0.71*	−0.20	−0.97**	0.50	1.62**	1.27**	0.82**	0.61*
2011	0	0	0	0	0	0	0	0	0	0

Ranges for all classifications are defined in Methods.

*P < 0.05, **P < 0.01.

**Table 6:** Lipid index classifications and prevalence of lipid conditions of civil aviators in China by age group, 2006–2011 (*N* = 305).

Age	*N*	TC, *N* (%)		TG, *N* (%)		HDL-C, *N* (%)	LDL-C, *N* (%)		Borderline high, *N* (%)	Hyperlipidemia, *N* (%)	Dyslipidemia, *N* (%)
		+	++	+	++	−	+	++			
21–30	255	23 (10.22)	2 (0.89)	29 (12.89)	8 (3.56)	50 (22.22)	62 (27.56)	10 (4.44)	62 (27.56)	64 (28.44)	114 (50.67)
31–40	1037	149 (14.37)	20 (1.93)	219 (21.12)	173 (16.68)	349 (33.65)	228 (21.99)	83 (8.00)	252 (24.30)	496 (47.83)	707 (68.18)
41–50	347	90 (25.94)	24 (6.92)	33 (9.51)	75 (21.61)	92 (26.51)	90 (25.94)	57 (16.43)	93 (26.80)	171 (49.28)	224 (64.55)
51–60	191	44 (23.04)	7 (3.67)	37 (19.37)	20 (10.47)	42 (21.99)	59 (30.89)	20 (10.47)	60 (31.41)	75 (39.27)	120 (62.83)

Ranges for all classifications are defined in Methods.
